# A novel microcapsule composite Spherulites Peony Superior Retinol mitigates UVB‐induced skin damage in vitro and in vivo

**DOI:** 10.1111/php.14078

**Published:** 2025-02-05

**Authors:** Jiejun Han, Rongyue Gong, Yuankun Liu, Tiangui Gong, Bin Wang, Laidi Zhang, Jiayue Chen

**Affiliations:** ^1^ MCL Skincare Ltd. Hangzhou China; ^2^ Research and Innovation Lab Hangzhou Shiguang Xinya Biotechnology Co., Ltd. Hangzhou China

**Keywords:** DNA damage repair, fibroblast, mast cell, Spherulites Peony Superior Retinol, UVB

## Abstract

Skin serves as our outermost barrier, protecting our bodies from various environmental damages. Increasing research has revealed that UVB is a primary factor for extrinsic aging. This study explored the role of a novel microcapsule composite Spherulites Peony Superior Retinol (SPSR) on skin damage induced by UVB. SPSR exhibited a capacity to eliminate UVB‐induced ROS. By measurement of cyclobutane pyrimidine dimers (CPD) and comet assay, the results implied that SPSR mitigates DNA damage from oxidative damage caused by UVB. In addition, UVB radiation typically leads to an increase in inflammatory factors within the skin. Decreased gene expressions of interleukin‐1α and TNF‐α have been observed in HaCaT cells. Moreover, a decreased gene expression of extracellular matrix (ECM)‐related protein, including fibronectin (*FN1*), *Col1A1*, and *Col3A1* caused by UVB was mitigated by SPSR. Furthermore, the clinical trials with 30 volunteers confirmed the significant relief and antiwrinkle effects of the cosmetic formulation containing 0.1% SPSR. These findings implied the promising potential of SPSR as a comprehensive solution for combating the detrimental effects of UVB exposure and maintaining skin health.

## Graphical abstract

This study explored the role of a novel microcapsule composite Spherulites Peony Superior Retinol (SPSR) on skin damage induced by UVB. The results of in vitro and human efficacy assays further demonstrate that SPSR is beneficial in replenishing skin hydration, promoting epidermal cell growth, soothing skin redness and sensitivity, reducing ROS generation, and promoting facial elasticity. These findings imply the promising potential of SPSR as a comprehensive solution for combating the detrimental effects of UVB exposure and maintaining skin health.

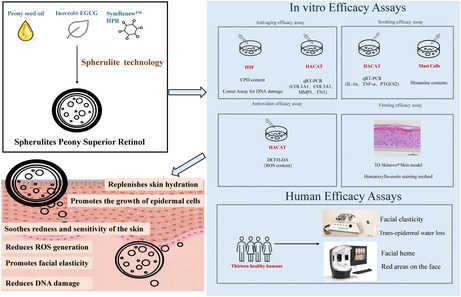

**Abbreviations**
3D Skin Model: Skinovo®‐Epi3D
COL1A1: collagen Type I alpha 1
COL3A1: collagen Type III alpha 1
CPD: cyclobutane pyrimidine dimers
DCFH‐DA: 2',7'‐dichlorodihydrofluorescein diacetate
DMEM: Dulbecco's Modified Eagle Medium
ECM: extracellular matrix
EGCG: epigallocatechin 3‐gallate
ELISA: enzyme‐linked immunosorbent assay
FN1: fibronectin 1
HE: hematoxylin and eosin
HPR: hydroxypinacolone retinoate
IL‐1α: interleukin‐1 alpha
MMP1: matrix metalloproteinase 1
PTGES2: prostaglandin E synthase 2
qRT‐PCR: quantitative real‐time polymerase chain reaction
ROS: reactive oxygen species
SPSR: spherulites peony superior retinol
TEWL: transepidermal water loss
TNF‐α: tumor necrosis factor alpha
UVB: ultraviolet B
WST‐8: sodium 4‐[3‐(2‐methoxy‐4‐nitrophenyl)‐2‐(4‐nitrophenyl)‐2H‐tetrazol‐3‐ium‐5‐yl]‐1,3‐benzenedisulfonate

## INTRODUCTION

The skin, being the largest organ of the human body, serves as a protective barrier against external damage. However, aging skin tends to exhibit a decline in its protective capabilities. Skin aging is mainly attributed to two main categories, intrinsic and extrinsic aging.^1^ Intrinsic aging is also regarded as chronological aging, which is primarily associated with factors such as genetics and cellular functional decline, whereas extrinsic aging is influenced by external environmental factors including aerial pollutants and ultraviolet (UV) radiation.^2^, ^3^ Extrinsic aging is primarily associated with UV exposure, also known as photoaging.^4^

Ultraviolet irradiation is primarily divided into UVA (320–400 nm) and UVB (280–320 nm). Among these, UVB radiation has been found to exert a greater impact on skin damage due to its shorter wavelength that makes it more readily absorbed by the superficial layers of the skin. UVB radiation can induce a range of adverse effects, including sunburn, pigmentation disorders, and skin cancer, highlighting the significance of understanding its effects for the prevention and treatment of skin damage.^4^, ^5^ Ravanat et al.^6^ revealed that UVB radiation can directly damage DNA, leading to disruption of repair mechanisms in the cell nucleus, thereby increasing the risk of skin diseases. The main indicator of DNA damage caused by UVB is the presence of photoproducts within the DNA, particularly the formation of bipyrimidine photoproducts such as cyclobutane pyrimidine dimers (CPDs) and 6–4 photoproducts (6‐4PPs).^7^ The accumulation of these photoproducts and any deficiencies in their repair would cause skin carcinogenesis.^8^ Detecting the quantity of the photoproducts serves as a significant indicator to assess the extent of DNA damage induced by UVB radiation. Numerous studies have demonstrated that the accumulation of reactive oxygen species (ROS) triggered by UVB could lead to DNA oxidation, including oxidation of single nucleotides, damage to bases, and other types of lesions.^7^, ^9^ Additionally, UVB‐induced ROS generation leads to an increased expression of MMPs via the AP‐1 (activator protein‐1) pathway, resulting in structural and functional damage to skin tissue and accelerating the aging process.

Microencapsulation technology has been widely employed in cosmetics fields, involving enclosing active ingredients within microscopic spheres to enhance stability, efficacy, and targeted delivery.^10^ It protects active ingredients from degradation and optimizes their release for prolonged periods, which is in favor of enhancing the effects in skincare. Spherulite contains 10–1000 surfactant bi‐layers, the characteristic of hydrodispersible/lipodispersible spherulite making it sure to well‐encapsulate both lipophilic and hydrophilic actives. It is a unique and flexible technology with a CNRS (Center National de la Recherche Scientifique) patent, suitable for various product formulations since it has the stability of the encapsulation technology. Moreover, spherulite technology can adhere to the surface layer of the skin, optimize the permeability, diffuse through the epidermis to the dermis, penetrate the active ingredients deeply into the skin, and control the release of the active for a long‐lasting effect.^11^

Tree peony (*Paeonia suffruticosa*) seed oil is rich in unsaturated fatty acids like α‐linolenic acid (ALA, >38%) that are beneficial for anti‐wrinkle, soothing, and whitening effects.^12^, ^13^ Epigallocatechin 3‐gallate (EGCG) from green tea boasts potent antioxidant properties, combating inflammation, aging, cancer, and obesity. It scavenges free radicals and protects against DNA damage from UV radiation.^14^, ^15^ Another antiaging ingredient, Hydroxypinacolone Retinoate (HPR), a retinoid derivative, possesses more stable activity with less skin irritation compared to retinol.^16^ Hence, a complex of peony seed oil, EGCG, and HPR encapsulated with Spherulite has the potential to be a potent antiaging compound with enhanced efficacy and minimal irritation.

In the previous patent, we introduced a novel microencapsulated active compound SPSR, comprising peony seed oil, EGCG, and HPR. These components collectively target key mechanisms underlying photodamage repair. SPSR provides a comprehensive approach to addressing the consequences of UV‐induced skin damage by alleviating oxidative stress and supporting the restoration of skin's structural integrity, highlighting its potential as a multifunctional agent for skin repair and recovery.

## MATERIALS AND METHODS

### Chemicals and regents

The patented ingredient Spherulites Peony Superior Retinol (SPSR; patent number, CN115887246A) was composed of 7.5% peony seed oil, 0.1% epigallocatechin gallatyl glucoside (EGCG), and 0.5% hydroxypinacolone retinoate (HPR) encapsulated by Spherulite (Givaudan, France) which is a microcapsulation technology characterized by a multi‐layered vesicle structure. Before treatments, the SPSR solution at different concentrations was filtered with a 0.22 μm filter (Jinteng Technology Co., Ltd.).

### Cells culture and treatment

Human immortalized keratinocytes (HaCaT) cell line and human fibroblast (HSF) were obtained from Beyotime. These cells were grown in culture medium DMEM supplemented with 10% fetal bovine serum and 100 units/mL penicillin/streptomycin (Gibco) at 5% CO_2_, 37°C. The culture medium was changed every 2 days. Cells were cultured in a complete serum medium in a 6‐well plate for 24 h. The SPSR of 0.01%, 0.005%, and 0.001% was added in a serum‐free DMEM medium, respectively. The cells in the logarithmic growth phase were seeded into a six‐well cell culture plate at a density of 5 × 10^5^/well. After the cells adhered for 24 h, the sample group and the model group were stimulated with UVB (60 mJ/cm^2^, UV07‐II, Licheng instrument Co., Ltd.). After stimulation, SPSR was added to the cell culture plate of the sample groups and incubated for 24 h. The control group (referred to no SPSR addition or UVB irradiation) and the model group (referred to UVB irradiation without SPSR addition for 24 h) were set up simultaneously using serum‐free DMEM medium for 24 h before harvest.

### Cell viability assay

Cell viability assay was carried out by WST‐8 assay (CCK‐8, Dojindo Chemical Technology Co. Ltd.). Briefly, cells were cultured in a 96‐well plate (8000 cells/well) in corresponding complete medium (100 μL) at 5% CO_2_, 37°C for 24 h. Subsequently, 100 μL of fresh complete culture medium containing specific concentrations of SPSR (0.001%, 0.005%, 0.01%, 0.05%, 0.1%, and 0.5%) was used to refresh the medium in each well, while the control group received 100 μL of fresh complete culture medium without SPSR. After an additional 24 h culture, 10 μL CCK‐8 solution was added to each well. Then, after 1 h incubation, the viability of the cells in each well was determined by measuring the absorbance at 450 nm with a microplate reader (Thermo Fisher). Cell viability was calculated using the following equation:

$$\begin{array}{c} \text{Cell viability}\;\left(\%\right)\\ =\frac{\mathrm{OD}\;\text{of treatment group}-\mathrm{OD}\;\text{of control group}}{\mathrm{OD}\;\text{of control}}\times 100. \end{array}$$

### Reactive oxygen species assay

Reactive oxygen species (ROS) content was measured using 2′,7′‐dichloro‐dihydrofluorescein diacetate (DCFH‐DA). Briefly, the cells from different treatments were washed with phosphate‐buffered saline (PBS) three times, diluted DCFH‐DA with a serum‐free culture medium at 1:1000 to a final concentration of 10 μM. Cells were collected and suspended in diluted DCFH‐DA and incubated at 5% CO_2_, 37°C for 20 min. After washing off redundant fluorescent probes, the fluorescence values in the cells were determined using a microplate reader (Thermo Fisher) with an excitation and emission wavelength of approximately 485 nm and 528 nm, respectively. The contents of ROS were calculated by the average fluorescence intensity.

### The measurement of DNA damage

#### Measurement of CPD

To assess the formation of CPDs (Detection of Cyclobutane Pyrimidine Dimers) after UVB radiation exposure, the following method was employed. Genomic DNA from cell samples was extracted using a DNA extraction kit (Tiangen biochemical Technology Co., Ltd.). By incubating the sample at 95°C for 10 min and rapidly cooling it on ice for 10 min, the DNA sample was converted into single‐stranded DNA, diluted to 4 μg/mL in cold TE buffer. The enzyme‐linked immunosorbent assay was performed by following the instructions of the assay kit manufacturer (Cell Biolabs).

#### DNA damage comet assay

The Comet Assay Kit (Cell Biolabs) was used to detect DNA breaks in cultured cells. DNA damage was assessed by denaturing DNA with an alkali solution for single‐strand breaks or a neutral solution for double‐strand breaks. Negatively charged DNA fragments migrated toward the anode. Briefly, cells were mixed with OxiSelect™ Comet Agarose at 37°C to a density of 1 × 10^4^ cells/mL. Then, 75 μL of the agarose/cell mixture was transferred per well onto the OxiSelect™ Comet Slide. The cells were treated with lysis buffer and alkaline solution, and electrophoresis was conducted at 25 V for 25 min. The slides were stained with Vista Green DNA Dye at room temperature for 15 min and observed under a fluorescence microscope (ICX41, SOPTOP, China) at 200× magnification. ImageJ software (National Institutes of Health) was used to analyze 10 cells per sample to determine the average percentage of DNA damage.

### Real‐time quantitative PCR

Cellular RNA was extracted utilizing the EasyPure® RNA Kit (TransGen Biotech), followed by cDNA synthesis using the TransScript® One‐Step gDNA Removal and cDNA Synthesis SuperMix (TransGen Biotech). Subsequently, quantitative real‐time polymerase chain reaction (qRT‐PCR) was performed with a 20 μL reaction volume containing 1 μL of cDNA, 7 μL ultrapure water, 1μL forward primer, 1 μL reverse primer, and 10 μL Thunderbird SYBR qPCR Mix (TOQPS‐201, TOYOBO, Japan). The thermocycler parameters were 95°C for 30 s, followed by 40 cycles of 95°C for 10 s and 60°C for 30 s. To analyze the relative expression levels of target genes, the comparative CT (2^−ΔΔCT^) method was employed. The sequences of all primers used in this study are listed in Table 1. Real‐time qPCR was performed using a thermocycler QuantStudio5 instrument (Thermo Fisher). The primer sequences used in this study were listed in Table 1. β‐actin was used as the control gene for normalization.

**TABLE 1:** Primer sequences used for gene expression analysis.

Target genes	Forward primers (5′–3′)	Reverse primers (5′–3′)
TNF‐α	GAGGCCAAGCCCTGGTATG	CGGGCCGATTGATCTCAGC
IL‐1α	TGGTAGTAGCAACCAACGGGA	ACTTTGATTGAGGGCGTCATTC
PTGES2	CCTCATCAGCAAGCGACTCA	ATACACCGCCAAATCAGCGA
MMP1	AAAATTACACGCCAGATTTGCC	GGTGTGACATTACTCCAGAGTTG
FN1	CGGTGGCTGTCAGTCAAAG	AAACCTCGGCTTCCTCCATAA
COL1A1	TGTGCGATGACGTGATCTGTGA	CTTGGTCGGTGGGTGACTCTG
COL1A3	CTCTGCTTCATCCCACTATTATTT	TCCGCATAGGACTGACCAAGAT
β‐Actin	CGGGAAATCGTGCGTGAC	GGAAGGAAGGCTGGAAGAGTG

### The 3D recombinant epidermal thickness analysis

The 3D recombinant epidermal model (Skinovo®‐Epi3D; Regenovo Biotechnology Co., Ltd.) was resuscitated using special medium and incubated at 37°C, 5% CO_2_ in an incubator for 24 h. Different concentrations of SPSR (0.001%, 0.005%, and 0.01%) and serum‐free medium (control group) were added to Skinovo®‐Epi3D and incubated at 37°C, 5% CO_2_ for 24 h. After being washed with PBS two times, the 3D epidermal model was taken out, embedded, and sectioned with a paraffin slicing machine (Leica RM2235), and the sections were stained using Hematoxylin and eosin (HE) staining. The stained sections were photographed through a microscope (VTSE3S2000, Weihan Optoelectronic Technology Co., Ltd.). The thickness of Skinovo®‐Epi3D was measured by ImageJ (National Institutes of Health).

### Human study design

An in vivo study was performed at the Veminsyn R&D Center. A total of 30 Chinese women subjects aged between 25 and 60 years were selected and provided informed consent. The study followed a double‐blind, randomized, split‐face trial design. Participants applied a placebo cream to the left side of their face and the SPSR cream (containing 0.1% SPSR) to the right side twice daily (morning and night) for a continuous 28 days. Clinical assessments were conducted at three time points: Day 0 (baseline), Day 14, and Day 28.

Elasticity, erythema, and transepidermal water loss (TWEL) levels in the skin were assessed using a multi‐function skin tester (Courage + Khazaka electronic GmbH). Skin redness area was evaluated based on the VISIA‐CR (Canfield Scientific). The clinical test was approved by the ethics committee of Shanghai WEIPU Testing Technology Group Co., Ltd. on February 2, 2023 (ethics review approval document number: WP‐202302JC04).

### Statistical analysis

All measurements and data were performed in three independent experiments. Data are presented as mean ± standard deviation (SD). Results were subjected to one‐way ANOVA using GraphPad Prism 6.0 (GraphPad Software, Inc.), with *p < 0.05, **p  < 0.01, and ***p < 0.001 indicating statistically significant differences. Differences between the control and model group (UVB) were compared using Student's *t*‐test, with #*p* < 0.05, ^##^ *p* < 0.01, and ^###^ *p* < 0.001.

## RESULTS

### Cell viability evaluation

**FIGURE 1:**
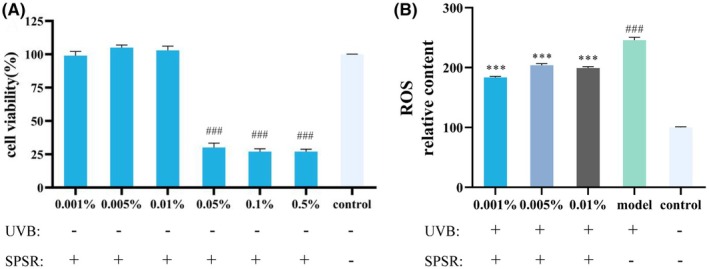
Effects of SPSR on cellular viability and ROS production in HaCaT cells. (A) Viability of HaCaT cells was measured by the WST‐8 assay following treatment with SPSR at various concentrations (0.001%, 0.005%, 0.01%, 0.05%, 0.1%, and 0.5%) for 24 h. Control referred to no SPSR addition. (B) Level of ROS was determined in cells treated with different concentrations of SPSR (0.001%, 0.005%, and 0.01%) after UVB irradiation (60 mJ/cm^2^) for 24 h. Control referred to neither SPSR addition nor UVB irradiation. Model referred to UVB irradiation (60 mJ/cm^2^) without SPSR addition. The ROS content of HaCaT cells was measured under an inverted fluorescence microscope after the addition of the DCFH‐DA probe and observed. The results were expressed as mean ± SD (*n* = 3). ^###^ *p*  < 0.001, compared to the control group; *p < 05, **p < 1, ***p < 1, compared to the model group.

To measure the cellular tolerance to SPSR, the effect of SPSR on the different cell viabilities of HaCaT cells and HSF cells was measured by the WST‐8 assay. The cells were exposed to the concentrations from 0.001% to 0.5% for 24 h. As the results shown in Figure 1A and Figure S1, SPSR at concentrations (0.001%, 0.005%, and 0.01%) did not affect the viability of the two types of cells. However, when the concentration of SPSR was above 0.05%, the cell viability was significantly decreased (Figure 1A–C). Consequently, the following cell experiments will be performed at the concentrations of 0.001%, 0.005%, and 0.01% SPSR.

### The accumulation of ROS in HaCaT cells

To investigate the role of SPSR on the accumulation of ROS induced by UVB, the level of ROS was quantified using the fluorescent probe DCFH‐DA. After UVB radiation (60 mJ/cm^2^), compared to the control group, the intracellular ROS level in the model group was significantly increased 1.5 times (Figure 1D; *p* < 0.001). Figure 1B clearly illustrated the comparative ROS levels before and after SPSR treatment. By contrast with UVB treatment, the addition of SPSR effectively reduced the ROS accumulation in HaCaT cells (Figure 1B, *p* < 0.001). Specifically, the levels of ROS in cells treated with SPSR at concentrations of 0.001%, 0.005%, and 0.01% were reduced by 183.55%, 203.88%, and 199.45% (Figure 1B; *p* < 0.001), respectively.

### Modulatory effects of SPSR on DNA damage repair in HaCaT cells

**FIGURE 2:**
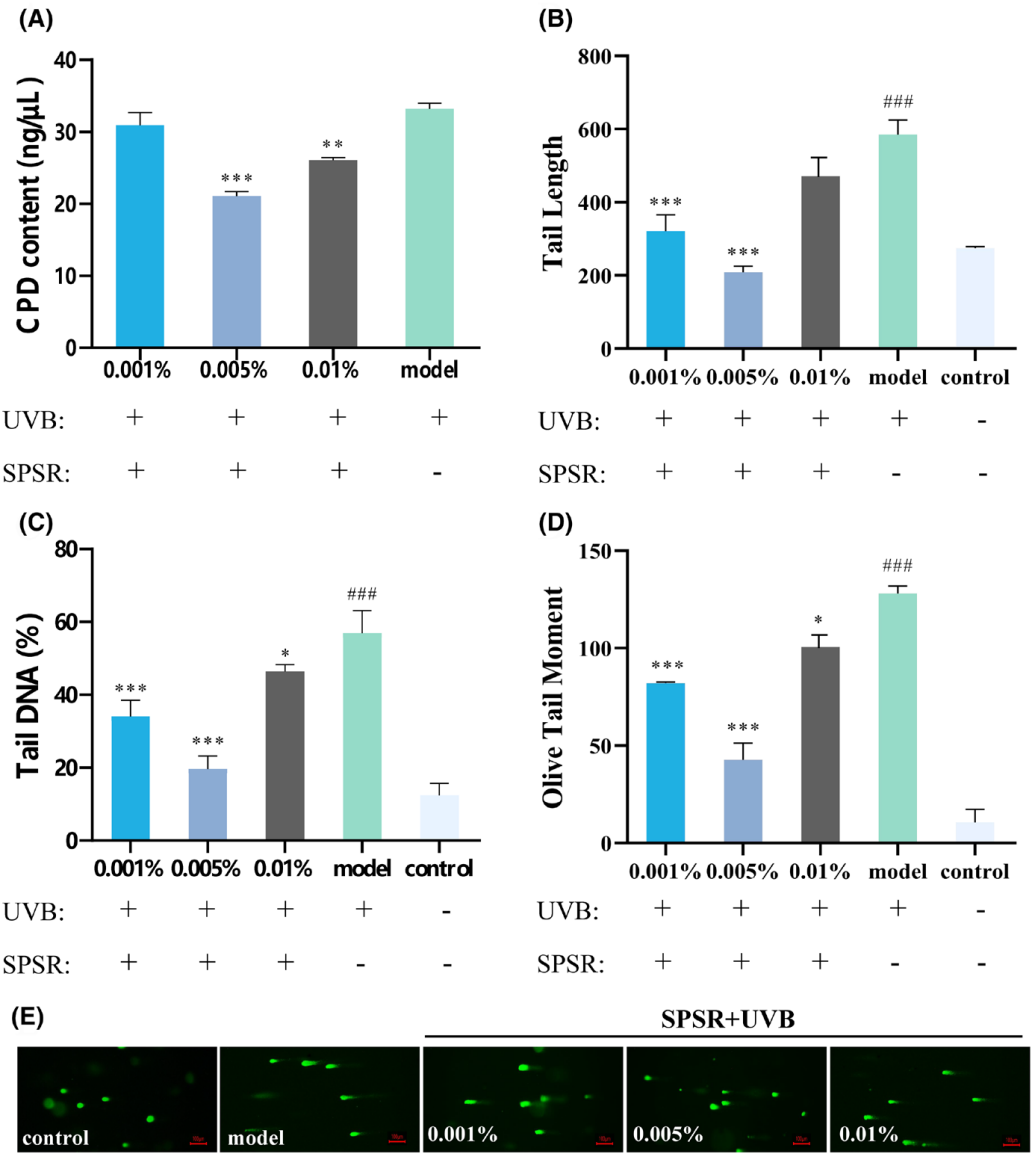
Effects of different concentrations of SPSR on UVB‐induced DNA damage. (A) CPD content was assayed by ELISA in HaCaT cells irradiated with 60 mJ/cm^2^ UVB and treated with SPSR (0.001%, 0.005%, and 0.01%) for 24‐h postirradiation. *N* = 3. To quantify the effects of SPSR on the DNA damage, the comet assay was performed in HaCaT cells. Parameters evaluated include tail length (B), tail DNA (C), and olive tail moment (D). Control referred to no SPSR addition or UVB irradiation. Model referred to UVB irradiation (60 mJ/cm^2^) without SPSR addition. Representative images of the comet experiments were shown (E). *N* = 10. The results were expressed as mean ± SD. ^###^ *p* < 0.001, compared to the control group; *p < 0.05, **p < 0.01, ***p < 0.001, compared to the model group.

The primary form of DNA damage induced by UVB radiation is the cyclobutane pyrimidine dimer (CPD), which impedes transcription and replication processes. CPD is also regarded as a specific marker for DNA damage detection caused by ultraviolet (UV) radiation. The removal of these dimers is crucial for preserving genomic integrity and function. In this study, we employed the ELISA assay to detect CPDs in UV‐damaged DNA. The CPD levels in the control group were extremely low (data not shown). As illustrated in Figure 2A, pretreatment with UVB (60 mJ/cm^2^), CPD contents were increased in HaCaT cells. After exposure to UVB, SPSR co‐cultured with HaCaT cells for 24 h; 0.005% and 0.01% of SPSR significantly reduced the CPD content by 36.7% and 19.3%, respectively (Figure 2A).

When a cellular DNA strand broke, its structure became compromised. The comet assay can evaluate the occurrence and repair process of DNA strand breaks following UV damage. Damaged DNA migrates under the influence of an electric field to form a tail resembling a comet. The length and density of this tail could be used to estimate the extent of DNA damage (Figure 2E). A longer tail and higher density typically indicate greater DNA fragmentation, which is indicative of more extensive DNA damage. The results illustrated that UVB treatment significantly enhanced the tail length (Figure 2B), tail DNA percentage (Figure 2D), and olive tail moment (Figure 2D; *p* < 0.001) compared to the control treatment. Following UVB radiation, the addition of SPSR at 0.005% and 0.01% resulted in a significant reduction in tail length (Figure 2B), tail DNA percentage (Figure 2C), and olive tail moment (Figure 2D) compared to the model group.

### Soothing benefits of SPSR

**FIGURE 3:**
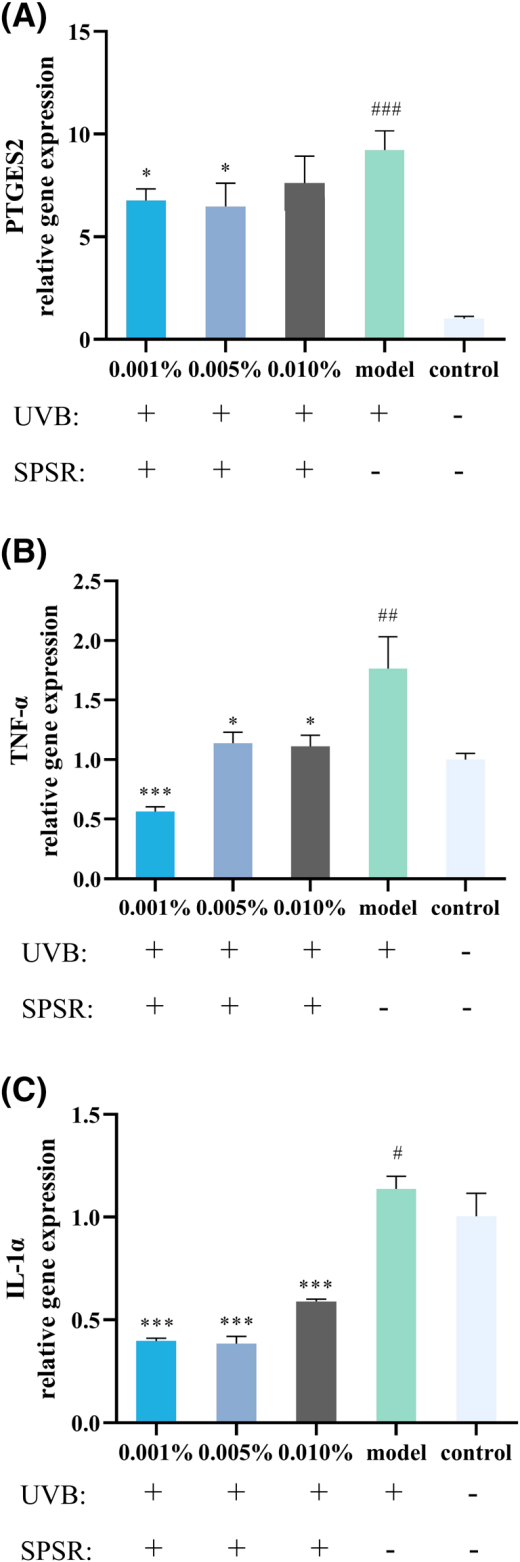
The soothing effect of SPSR. Real‐time qPCR was performed to analyze the regulatory effects of the samples on the expression of inflammatory factor genes, including *PTGES2* (A), *TNF‐α* (B), and *IL‐1α* (C). Control referred to no SPSR addition or UVB irradiation. Model referred to UVB irradiation (60 mJ/cm^2^) without SPSR addition. *N* = 3. The results were expressed as mean ± SD. ^#^ *p* < 0.05, ^##^ *p* < 0.01, ^###^ *p*  < 0.001, compared to the control group. *p < 0.05, **p < 0.01, ***p < 0.001, compared to the model group.

Prostaglandin E2 (PGE2) plays an important role in the skin, particularly in inflammation, immune modulation, and the repair process following skin damage. Prostaglandin E Synthase 2 (PTGES2) is one of the key enzymes in the synthesis of PGE2. For *PTGES2*, UVB treatment significantly enhanced its gene expression by 8.2 times compared to control (Figure 3A). Post‐UVB exposure, SPSR at concentrations of 0.001%, 0.005%, and 0.01% mitigated the UVB‐induced *PTGES2* gene expression, which was decreased by 19.0%, 35.2%, and 7.9%, respectively (Figure 3A).

TNF‐α belongs to the class of pro‐inflammatory cytokines that are involved in normal inflammatory and immune responses and can synergistically regulate the production of other cytokines, such as IL‐1α. We assessed the ameliorative effect of SPSR on the inflammation factors by qPCR. Compared to the model group, all SPSR‐treated groups (0.001%, 0.005%, and 0.01%) showed significant reductions in *TNF‐α* gene expression of 61.76%, 22.85%, and 24.76%, respectively (Figure 3B). Similarly, compared to the model group, SPSR at concentrations of 0.001%, 0.005%, and 0.01% reduced *IL‐1α* gene expression, with inhibition rates of 60.74%, 65.40%, and 55.69%, respectively (Figure 3C).

### Effects of SPSR on antiaging in HSF cells

**FIGURE 4:**
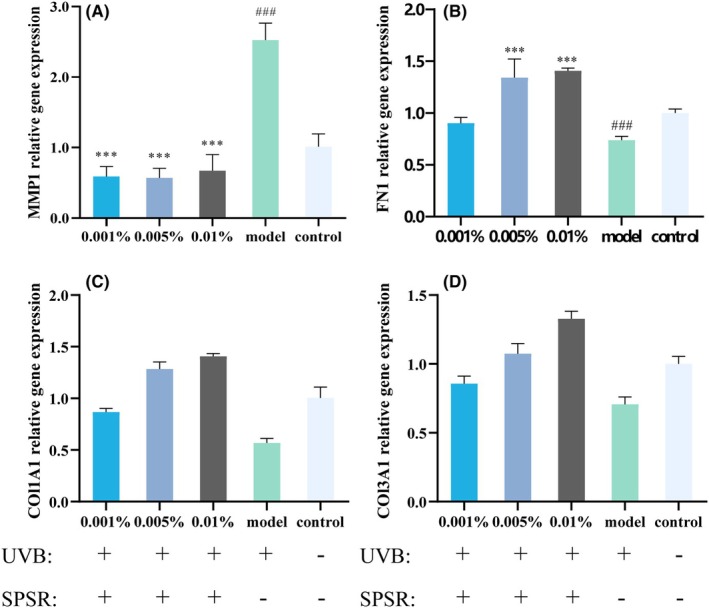
Evaluation of antiaging efficacy of SPSR in HSF cells. Real‐time qPCR analyzed the regulatory effects of the SPSR on the expression of ECM‐related genes, including *MMP1* (A), *FN1* (B), *COL1A1* (C), and *COL3A1* (D). Control referred to no SPSR addition or UVB irradiation. Model referred to UVB irradiation (60 mJ/cm^2^) without SPSR addition. *N* = 3. The results were expressed as mean ± SD. ^##^ *p* < 0.01, ^###^ *p* < 0.001, compared to the control group. *p < 0.05, **p < 0.01, ***p < 0.001, compared to the model group.

To evaluate the role of SPSR on ECM structure, the gene expressions of *MMP1*, *FN1*, *COL1A1*, and *COL3A1* were detected in HSF cells. Compared with the control treatment, the stimulation of UVB elevated the expression of *MMP1* by 152.5% (Figure 4A) while separately decreasing the expression of *FN1*, *COL1A1*, and *COL3A1* by 24.1%, 43.2%, and 29.4% (Figure 4B–D). In contrast with the model treatment, the addition of SPSR at the concentrations of 0.001%, 0.005%, and 0.01% after UVB exposure reduced the expression of *MMP1* (decreased by 76.7%, 77.4%, and 73.3%), upregulated the expression of *FN1* (), *COL1A1* (increased by 52.3%, 125.1%, and 146.9%), and *COL3A1* (increased by 21.1%, 52.1%, and 87.9%) (Figure 4B–D).

### Effects of SPSR on the thickness of epidermis

**FIGURE 5:**
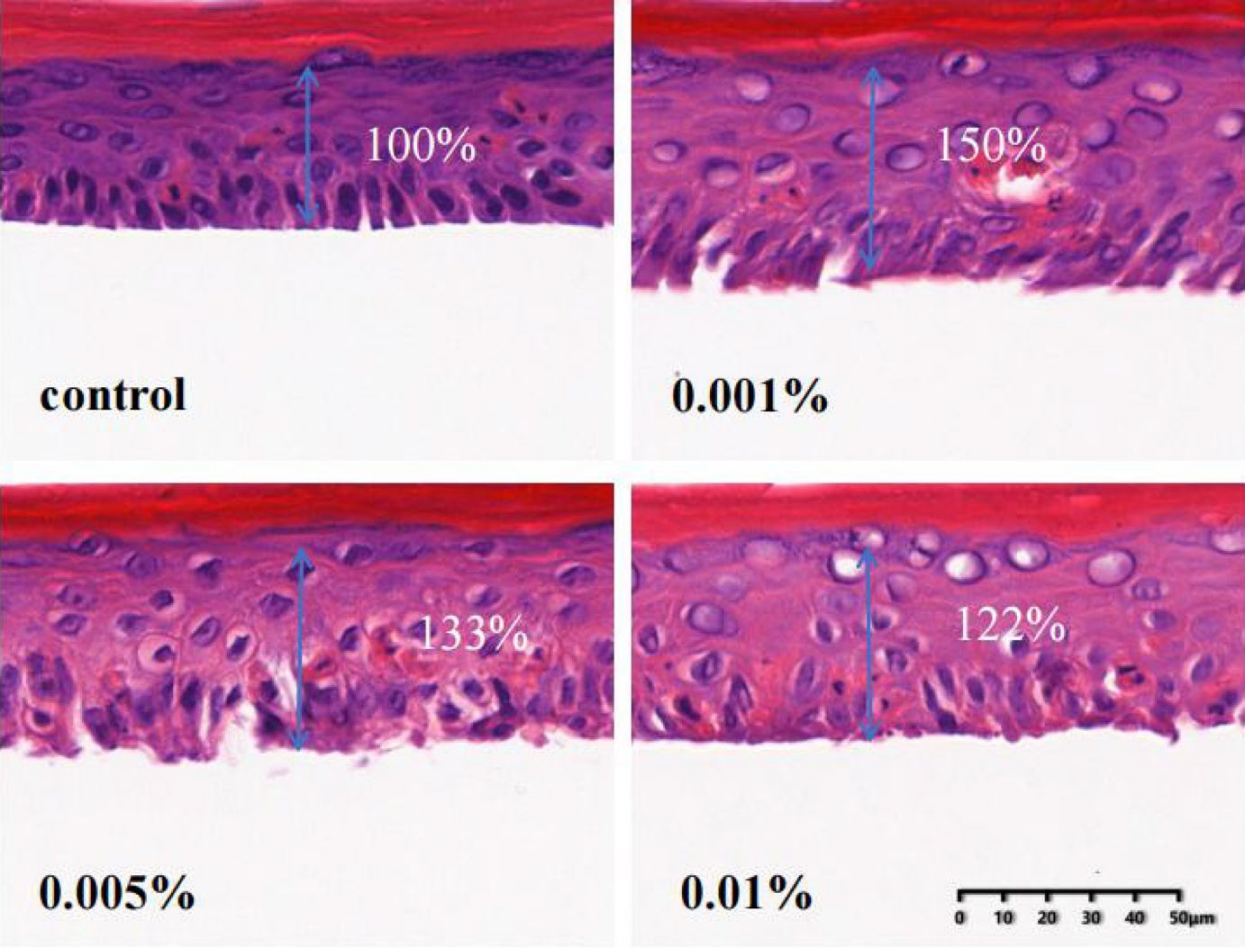
Histological evaluation of the effects of SPSR on the thickness of the epidermis through a 3D recombinant epidermal model. Skinovo®‐Epi3D models were treated with serum‐free medium (control) or SPSR at concentrations of 0.001%, 0.005%, and 0.01% for 24 h at 37°C with 5% CO₂. *N* = 3.

As individuals age, the skin tends to become thinner. To evaluate the changes in epidermal thickness, we utilized a recombinant 3D human epidermal model and conducted HE staining. Following a 24‐h treatment of the 3D epidermal model with varying concentrations (0.001%, 0.005%, and 0.01%) of SPSR, the images (Figure 5) showed a notable difference compared to the control group. Statistically, 0.001% and 0.005% of SPSR exhibited an increased epidermal thickness, with the epidermal thickness reaching 150% and 133% of the control group, respectively (Figure 5).

### Soothing and elasticity effects of SPSR on the skin in vivo

We performed a placebo trial experiment to evaluate the effect of SPSR as a cosmetic ingredient on antiaging and skin repair measurement of skin elasticity, transepidermal water loss (TWEL), skin erythema, and red area were recorded for both the placebo group and the tested group (cream containing 0.1% SPSR). Studies have shown that the first exposure to retinol in cosmetics needs to start at a concentration of 0.1%. To further demonstrate that the 0.1% concentration is appropriate, we performed a closed skin patch test (30 persons). The results showed that SPSR did not show a positive reaction at the 0.1% concentration, which means that this concentration does not cause skin irritation (Table S1). Therefore, we chose a concentration of 0.1% for the in vivo test in the subsequent experiments.

**FIGURE 6:**
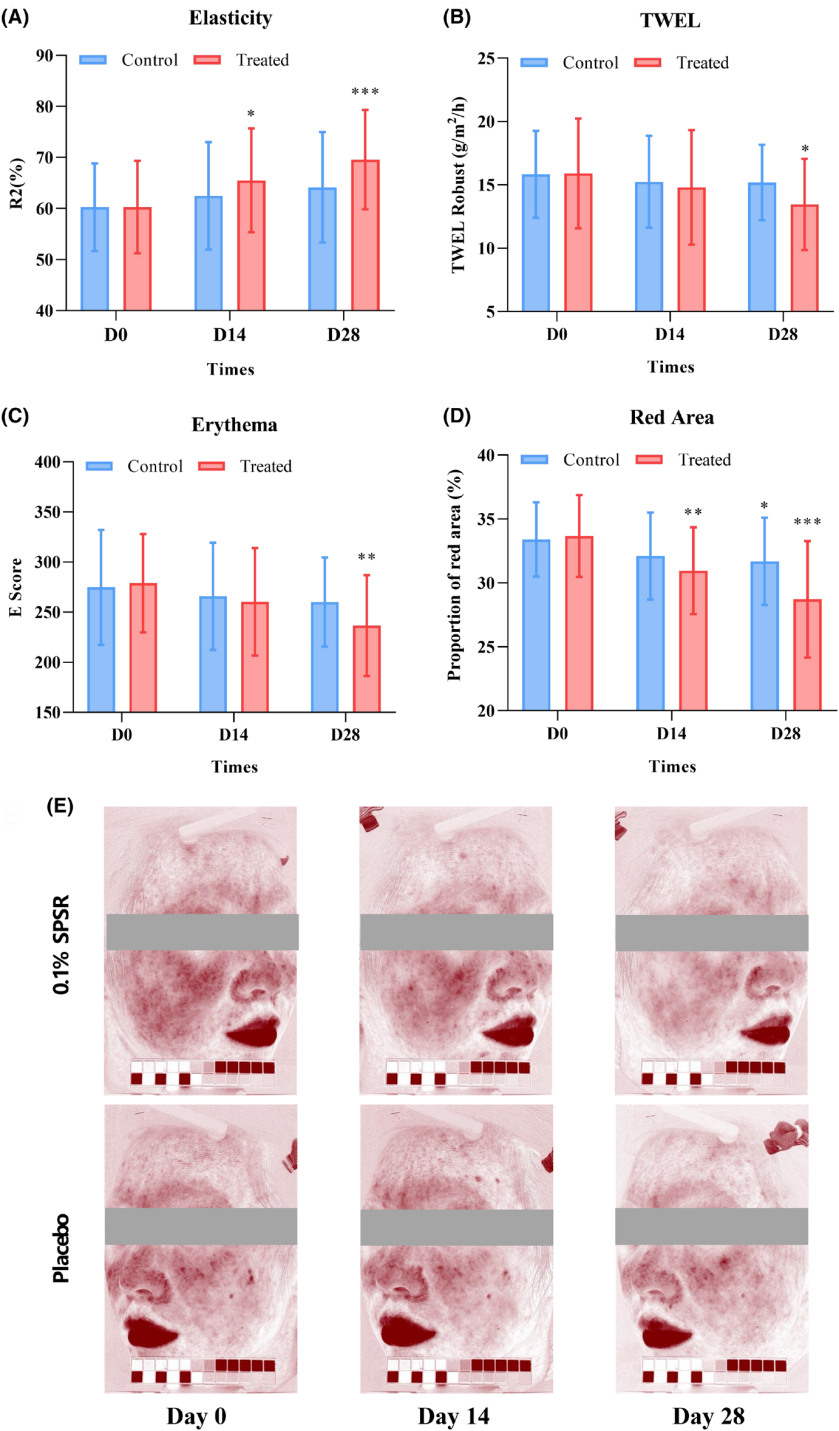
Results from the clinical test of the cream containing 0.1% SPSR. (A) Elasticity of skin. (B) Transepidermal water loss of skin. (C) Erythema of skin. (D) Red zone of skin. (E) Exemplary figure of the facial red area of the volunteers who participated in the SPSR clinical test at 0, 14, and 28 days. Participants applied a placebo cream to the left side of their face (Control) and the SPSR cream to the right side (Treated) twice daily (morning and night). *N* = 30. *p < 0.05, **p < 0.01, ***p < 0.001, compared to the placebo group.

In the placebo group, the initial skin elasticity measurement (Cutometer® MPA 580) was 60.25 ± 8.65, which increased to 62.49 ± 10.5 after 2 weeks and 64.16 ± 10.8 after 4 weeks. Conversely, in the SPSR‐treated group, the initial skin elasticity measurement was 60.29 ± 9.0 and increased to 65.52 ± 10.2 after 2 weeks of treatment and further to 69.59 ± 9.7 after 4 weeks (Figure 6A). Regarding TWEL (Tewameter® TM Hex), the placebo group initially recorded a measurement of 15.84 ± 3.4, which decreased to 15.2 ± 3.6 after 2 weeks and 15.20 ± 3.0 after 4 weeks. In contrast, for the tested group, the initial TWEL measurement was 15.911 ± 4.33, which decreased to 14.81 ± 4.5 after 2 weeks and 13.46 ± 3.6 after 4 weeks (Figure 6B).

Skin sensitivity changes, as indicated by skin erythema (Mexameter® MX 18) and the red area of the skin (VISIA CR), were evaluated initially and at 2 and 4 weeks following the application of the test product. In the placebo group, both erythema and the initial red area showed a trend of reduction over the course of the study. Specifically, erythema decreased from 274.82 ± 57.4 initially to 265.88 ± 53.4 after 2 weeks and remained at 260.21 ± 44.4 after 4 weeks, whereas the red area decreased from 33.41 ± 2.9 initially to 32.11 ± 3.4 after 2 weeks and 31.69 ± 3.4 after 4 weeks. Conversely, in the SPSR‐treated group, both erythema and the red area exhibited significant reductions. For instance, erythema decreased from 278.98 ± 49.1 initially to 260.41 ± 53.6 after 2 weeks and further declined to 236.65 ± 50.3 after 4 weeks. Similarly, the red area decreased from 33.68 ± 3.2 initially to 30.96 ± 3.4 after 2 weeks and further declined to 28.72 ± 4.56 after 4 weeks (Figure 6C,D,E).

## DISCUSSION

Skin aging represents a multifaceted process influenced by various intrinsic and extrinsic factors, with exposure to UV radiation playing a predominant role in extrinsic aging.^17^, ^18^ The daily exposure to UV, especially UVB, poses a significant threat to the integrity and function of the skin.^19^ Photo‐aged skin features as abnormal hyperkeratosis in the stratum corneum, a fragile epidermal barrier, and an irregular basal membrane with fine lines,^19^, ^20^ wrinkles, sagging, and uneven pigmentation. Simulating UVB‐induced skin damage is crucial for studying the underlying mechanisms of photoaging and evaluating potential interventions to alleviate these effects. Given these effects, interventions aimed at mitigating UVB‐induced skin damage are crucial for alleviating signs of aging. Extrinsic aging can be modulated through the adoption of effective skincare practices. Therefore, the identification of novel compounds and interventions capable of preventing photo‐damage holds promise in combating the signs of aging and maintaining skin youth. Previous studies have demonstrated the skin‐protective effects of single peony seed oil, epigallocatechin gallatyl glucoside (EGCG), and hydroxypinacolone retinoate (HPR), mainly associated with their abilities to scavenge reactive oxygen species (ROS) and inhibit inflammatory factors.^12^, ^14^, ^16^ However, these three active ingredients were unstable, making it difficult to directly incorporate them into cosmetics formulations. Previously, we made a novel microcapsule composite, Spherulites Peony Superior Retinol (SPSR; patent number, CN115887246A) comprising peony seed oil, EGCG, and HPR. In the present study, we investigated the mitigating effect of SPSR on UVB‐induced skin damage in vitro and in vivo.

Typically, UVB radiation instigates ROS within the skin, triggering oxidative stress which causes damage to cell membranes, lipids, and DNA, further exacerbating the aging process.^21^ Here, 60 mJ/cm^2^ UVB radiation increased the accumulation of ROS in HaCaT cells (Figure 1B). The formation of cyclobutane pyrimidine dimers (CPDs) represents one of the primary DNA photoproducts induced by UVB radiation. These dimers occur when UVB directly interacts with the DNA, causing structural distortions that disrupt normal DNA function.^22^, ^23^ After UVB radiation, ROS are generated indirectly through photosensitized reactions and contribute to oxidative DNA damage.^24^ The comet assay was generally used to evaluate DNA repair condition.^25^ Hence, the detection of CPD levels along with the comet assay provides a more comprehensive understanding of UV‐induced DNA damage and subsequent repair processes. Our results were consistent with previous findings, showing that UVB exposure led to an increase in CPD levels and elongation of DNA tails, as observed in the comet assay (Figure 2). These results collectively highlight that SPSR mitigates UVB‐induced DNA damage. The reduction in CPD levels and decreased DNA tail parameters suggest that SPSR effectively safeguards DNA. Since UVB irradiation was applied prior to the treatment with the active compound (SPSR), it is plausible that the cellular environment had already been significantly altered by the initial UVB exposure. This preconditioned state might have influenced the cellular response to subsequent SPSR treatment. Notably, at higher concentrations of SPSR (0.01%), the compound may exert an overwhelming effect on cellular processes, potentially interfering with the natural repair mechanisms activated by UVB‐induced damage. This could explain the observed reduction in efficacy at higher concentrations, suggesting that a delicate balance between the UVB‐induced stress response and SPSR treatment is crucial for optimal outcomes.

Research has shown that UVB radiation induces an inflammatory response in the skin, marked by an increase in inflammatory factors such as IL‐1 and IL‐6.^26^ Similar to previous research findings, UVB irradiation increased the levels of TNF‐α and IL‐1α (Figure 3). This inflammatory cascade triggers the upregulation of matrix metalloproteinases (MMPs), enzymes responsible for degrading the extracellular matrix (ECM) components like collagen and elastin. Thus, the skin loses its elasticity and resilience, contributing to the aging process. However, upon the addition of SPSR, we observed a notable reduction in inflammatory factors (TNF‐α, IL‐1α) and MMP1 expression levels. Consequently, in HSF cells, SPSR addition contributes to an increase in type I collagen (COL1A1) and type III collagen (COL3A1). This suggests that SPSR may exert its antiaging effects by mitigating the inflammatory response and suppressing MMPs. Therefore, the findings underscore the potential of SPSR as a promising skincare ingredient for combating UVB‐induced skin aging. Further elucidation of the underlying mechanisms and long‐term clinical studies will provide valuable insights into its efficacy and safety profile in skincare formulations.

The human epidermis serves as the initial line of defense against environmental stressors, including UV radiation. The gradual thinning of the epidermal layer is a hallmark of skin aging,^27^ contributing to increased vulnerability to external insults and decreased skin integrity. Therefore, strategies aimed at preserving epidermal thickness and enhancing cellular vitality are paramount in combating the aging process. The utilization of 3D skin models represents a significant advancement in dermatological research, offering a powerful tool for epidermis thickness.^28^ In the present study, the results reveal the efficacy of SPSR in promoting epidermal thickening (Figure 5), a crucial aspect of combating skin aging and maintaining skin integrity by utilizing 3D skin models.

To enhance the credibility of the research findings in vitro, we conducted a clinical trial experiment. Studies have shown that the first exposure to retinol in cosmetics needs to start at a concentration of 0.1% and then gradually helps the skin to establish tolerance. To further demonstrate the role of SRSP in skin protection, a cream containing 0.1% SPSR was used after a closed skin patch test (Figure 6 and Table S1). The test product exhibited not only a noteworthy decrease in TWEL but also erythema and red area after 4 weeks of use compared to the initial measurement (Figure 6B–E), implying the role of SPSR in soothing skin. Furthermore, the trial results also demonstrated a significant increase in skin elasticity levels, which is advantageous for skin antiaging.

In conclusion, our study introduces a novel microcapsule, termed Spherulites Peony Superior Retinol (SPSR), designed to encapsulate peony seed oil, EGCG, and HPR, renowned for their antioxidant and anti‐inflammatory properties. Through the Spherulite technology, the stability of these active ingredients was enhanced. Our findings indicate that SPSR could effectively scavenge free radicals, reducing DNA damage and thus mitigating the production of pro‐inflammatory cytokines such as TNF‐α and IL‐1α. Moreover, our investigation indicated the beneficial impact of SPSR on the downregulation of *MMP1*. By inhibiting the expression of MMP1, the degradation of collagens and other proteins within the ECM was prevented, thereby exerting its antiaging effect. Furthermore, clinical trials have validated these outcomes, demonstrating that skincare products containing SPSR enhance skin elasticity, diminish erythema, and bolster the skin's barrier function as well.

## Supporting information


Data S1.

## Data Availability

Data sharing is not applicable to this article as no new data were created or analyzed in this study.
