# Supplementary material for: A novel microcapsule composite Spherulites Peony Superior Retinol mitigates UVB‐induced skin damage in vitro and in vivo
Source: Photochem Photobiol. 2025 Feb 5;101(6):1468–79. doi: 10.1111/php.14078 (PMC12621075; doi:10.1111/php.14078)
Supplement: Supplementary file 1 — Data S1. [file PHP-101-1468-s001.docx]

**Supplementary Materials**

**Table S1** Results of human skin patch test.

| Groups | Participants  (Person) | Times  (hour) | The number of different skin reactions in patch test | | | | |
| --- | --- | --- | --- | --- | --- | --- | --- |
|  |  |  | 0 (-) | 1 (±) | 2 (+) | 3 (++) | 4 (+++) |
| NC | 30 | 0.5 | 27 | 3 | 0 | 0 | 0 |
| BC | 30 | 0.5 | 27 | 3 | 0 | 0 | 0 |
| 0.1% SPSR | 30 | 0.5 | 25 | 5 | 0 | 0 | 0 |
| NC | 30 | 6 | 29 | 1 | 0 | 0 | 0 |
| BC | 30 | 6 | 29 | 1 | 0 | 0 | 0 |
| 0.1% SPSR | 30 | 6 | 28 | 2 | 0 | 0 | 0 |
| NC | 30 | 12 | 30 | 0 | 0 | 0 | 0 |
| BC | 30 | 12 | 30 | 0 | 0 | 0 | 0 |
| 0.1% SPSR | 30 | 12 | 30 | 0 | 0 | 0 | 0 |
| NC | 30 | 24 | 30 | 0 | 0 | 0 | 0 |
| BC | 30 | 24 | 30 | 0 | 0 | 0 | 0 |
| 0.1% SPSR | 30 | 24 | 30 | 0 | 0 | 0 | 0 |

The results of human patch test showed that 28 cases were negative, and 2 cases were suspicious after 0.5 h of removal of the test substance. After 6 hours of removal of the test substance, 29 cases were negative and 1 case was suspicious. After 12 hours of removal of the test substance, there were 30 negative cases and 0 suspicious reaction. After 24 hours of removal of the test substance, there were 30 negative cases and 0 suspicious reaction. In summary, there was no positive reaction in the skin closed patch test (30 persons).


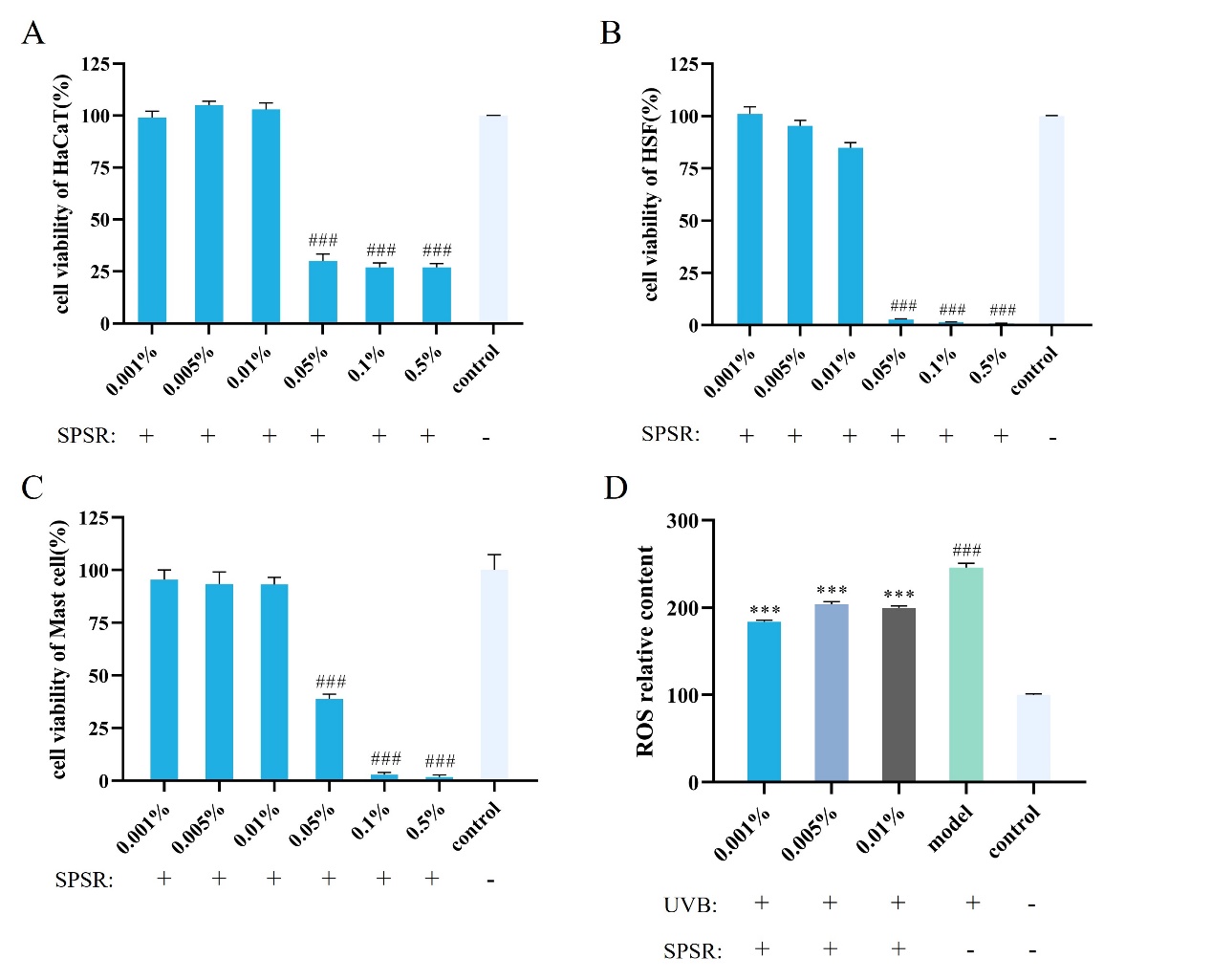


**Supplementary Figure 1.** Viability of HSF cells treated with SPSR at various concentrations (0.001%, 0.005%, 0.01%, 0.05%, 0.1% and 0.5%) for 24 hours. Control referred to no SPSR addition. The results were expressed as mean ± SD (n = 3). ### P < 0.001, compared to control group.
